# 

**Published:** 1898-11-05

**Authors:** 


**Notices of Births, Deaths, and Marriages** are inserted in this column at a charge of 2s. 6d. for an announcement not exceeding four lines.

### NOTICE TO CORRESPONDENTS.

All MS., Letters, Books for Review, and other matters intended for the Editor should be addressed The Editor, "The Hospital," The Hospital Building, 28 & 29, Southampton Street, Strand, W.C.

The Editor cannot uncertake to return rejected MS., even when ccompanied by stamped directed envelope.

**Notice to Advertisers and Subscribers.**

All Advertisements, Orders for Copies of The Hospital, and Business Communications should be addressed to **THE MANAGER (Not to the Editor),** The Hospital, The Hospital Building, 28 & 29, Southampton Street, Strand, London, W.C.

The Cost of Subscription, post free, is as follows:—Per week, 2½d.; per quarter, 2s. 8d.; per half-year, 5s. 3d.; per annum, 10s. 6d. Foreign:—Per annum, 15s. 2d., payable, in all cases, in advance.

**NOTICE.—Vol. XXIV. of "The Hospital" (April, 1898, to September, 1898), in handsome cloth binding, is now on sale at the Office of this Journal, price 7s. 6d. Cases for binding the Numbers contained in this Volume 1s. 6d. Index to Vol. XXIV., 2½d., post free.**

**The Monthly Part for November is now ready. Price 6d., by post 8d.**

### BOOKS RECEIVED.

Cassell and Co.

"Origin and Progress of Renal Surgery." Being the Hunterian Lectures for 1898. By Henry Morris, M.A., M.B., F.R.C.S.

"Diseases of the Skin." By Malcolm Morris.

Henry J. Glaisher.

"The Study of the Hand." By Edward Blake, M.D.

William Blackwood and Sons.

"Windyhough." By Graham Travers.

George Bell and Sons.

"Humane Science Lectures."

Editor Publishing Co.

"Cyclic Law." By Thomas M. Reed, M.D.

**Periodicals and Pamphlets.**—Nature Notes, Leeds Hospital Magazine, Woman's Signal, Information Gazette, Literary Digest, Medical Record, Medical Press, Melbourne Sun, English Illustrated Magazine, Guyoscope, Asylum News, Medical Temperance Review, Charity Organisation Review, Trained Nurse Review, Leisure Hour, Bibliographie Medicale, Guy's Hospital Gazette, Boy's Own Paper, Sunday at Home, Woman, Westminster Review, London, Commerce, G. O. P. Monthly Supplement.

### The Student of Medicine.

### APPOINTMENTS.

A. H. F. Barbour, M.D., F.R.C.P.Edin., appointed Assistant Physician to the Edinburgh Royal Maternity and Simpson Memorial Hospital. J. M. Beattie, M.B., C.M.Edin., appointed Assistant Professor of Pathology at the University of Edinburgh. J. Blamey, M.R.C.S.Eng., L.S.A., appointed Medical Officer for the Mylor District of the Falmouth Union. C. E. S Brettingham, L.F.P.S.Glasg., L.A.H.D. and D.P.H.R.C.S.I., appointed Medical Officer for the Third District of the Yeovil Union. E. C. Bridges, M.B., B S.Durh., appointed Physician-in-Ordinary to the Infirmary for Consumption, Margaret Street, Cavendish Square, W. A. H. Burgess, M.B., B.Ch.Vict., appointed Second Assistant Medical Officer to the Crumpsall Workhouse, Manchester. W. B. De Jersey, M.B., B.C., L.R.C.P.Lond., appointed Honorary Assistant Medical Officer to the Surrey County Hospital. A. R. Down, L.R.C.P.Lond., L S.A., appointed Medical Officer for the Bampton District of the Tiverton Union. A. F. Evison, appointed Medical Officer for the Second District of the North Witchford Union. S. C. C. Fenwick, M.R.C.S., L.R.C.P., appointed Medical Officer for the No. 10 District of the Lambeth Union. E. E. Goodbody, M.B.Dub., appointed Medical Officer for the Great Bardsfield District of the Dunmow Union. F. M. Graham, L.R.C.P., L.R.C.S.Edin, appointed Medical Officer for the Fifth District of the Drayton Union. Dr. Jolley, appointed Medical Officer for the No. 6 District of the Bridgwater Union. Dr. Kitchin, appointed Medical Officer for the Box District of the Chippenham Union. Dr. B. Locking, appointed to the Honorary Medical Staff of the Napier Hospital, Hawke's Bay, New Zealand. F. P. Mackie, M.R.C.S.Eng., L.R.C.P.Lond., appointed Junior Assistant Medical Officer to Salop and Montgomery Asylum, near Shrewsbury. J. A. MacLaren, M.B, appointed Senior Assistant Medical Officer to the Crumpsall Workhouse, Manchester. A. T. Morgan, M.D.Brux., L.S.A., appointed Medical Officer for the No. 4 District of the Bristol Union. D. C. Muir, M.D.Glasg., appointed Medical Officer of Health to the Abertillery Urban District Council. J. W. Mulligan, M.D.R.U.I., M.Ch, appointed Medical Officer for the Abersychan Central District of the Pontypool Union. A. C. Parsons, M.R.C.S.Eng., L.R.C.P.Lond, appointed House Surgeon to the Victoria Hospital for Children, Chelsea.

Second Edition. Price 6d.,
THE SCHOTT TREATMENT
FOR
CHRONIC HEART DISEASES.
By RICHARD GREENE, F.R.C.P.Ed.
London: The Scientific Press, Ltd., 28 & 29, Southampton Street, Strand, W.C.

